# The soft X-ray and XUV split-and-delay unit at beamlines FL23/24 at FLASH2

**DOI:** 10.1107/S1600577523000395

**Published:** 2023-02-15

**Authors:** Matthias Dreimann, Frank Wahlert, Dennis Eckermann, Felix Rosenthal, Sebastian Roling, Tobias Reiker, Marion Kuhlmann, Sven Toleikis, Maciej Brachmanski, Rolf Treusch, Elke Plönjes, Björn Siemer, Helmut Zacharias

**Affiliations:** a Center for Soft Nanoscience, Westfälische Wilhelms-Universität Münster, Busso-Peus-Str. 10, 48149 Münster, Germany; b Physikalisches Institut, Westfälische Wilhelms-Universität Münster, Wilhelm-Klemm-Str. 10, 48149 Münster, Germany; c Deutsches Elektronen-Synchrotron DESY, Notkestr. 85, 22607 Hamburg, Germany; University of Tokyo, Japan

**Keywords:** time-resolved pump–probe, XUV, soft X-rays, free-electron laser

## Abstract

A split-and-delay unit for the extreme ultraviolet and soft X-ray spectral regions has been built which enables time-resolved experiments at beamlines FL23 and FL24 at the Free-electron LASer in Hamburg (FLASH). Geometric wavefront splitting at a sharp edge of a beam splitting mirror is applied to split the incoming soft X-ray pulse into two beams. Ni and Pt coatings at grazing incidence angles have been chosen in order to cover the whole spectral range of FLASH2 and beyond, up to *h*ν = 1800 eV. In the variable beam path with a grazing incidence angle of ϑ_d_ = 1.8°, the total transmission (*T*) ranges are of the order of 0.48 < *T* < 0.84 for *h*ν < 100 eV and *T* > 0.50 for 100 eV < *h*ν < 650 eV with the Ni coating, and *T* > 0.06 for *h*ν < 1800 eV for the Pt coating. For a fixed beam path with a grazing incidence angle of ϑ_f_ = 1.3°, a transmission of *T* > 0.61 with the Ni coating and *T* > 0.23 with a Pt coating is achieved. Soft X-ray pump/soft X-ray probe experiments are possible within a delay range of −5 ps < Δ*t* < +18 ps with a nominal time resolution of *t*_r_ = 66 as and a measured timing jitter of *t*_j_ = 121 ± 2 as. First experiments with the split-and-delay unit determined the averaged coherence time of FLASH2 to be τ_c_ = 1.75 fs at λ = 8 nm, measured at a purposely reduced coherence of the free-electron laser.

## Abstract 2

The properties of the recently installed split-and-delay unit at beamlines FL23 and FL24 at FLASH2 are presented. Its operational range, performance parameters and results of a first experiment are described.

## 1. Introduction

Time-resolved pump/probe experiments in the extreme ultraviolet (XUV) and X-ray spectral regions performed with free-electron lasers (FELs) contribute to a fundamental understanding of the femtosecond dynamics of high-energetic processes of a large variety of physical systems. The requirements to perform such experiments are demanding and the key parameter to access the dynamics under investigation is the time evolution of the system. In the past many experiments have been carried out to examine the dynamics in atoms (Radcliffe *et al.*, 2012 ▸; Ding *et al.*, 2019 ▸), molecules (Krikunova *et al.*, 2012*a* ▸; Schnorr *et al.*, 2014 ▸; Liekhus-Schmaltz *et al.*, 2015 ▸; Lehmann *et al.*, 2016 ▸; Hollstein *et al.*, 2019 ▸; Rebholz *et al.*, 2021 ▸), clusters (Krikunova *et al.*, 2012*b* ▸; Ferguson *et al.*, 2016 ▸; Oelze *et al.*, 2017 ▸; Asmussen *et al.*, 2022 ▸), hot dense matter (Zastrau *et al.*, 2014 ▸) and condensed matter systems (Inoue *et al.*, 2016 ▸; Pardini *et al.*, 2018 ▸; Roseker *et al.*, 2018 ▸; Vinko *et al.*, 2020 ▸; Liu *et al.*, 2021 ▸). Results on time-resolved imaging (Günther *et al.*, 2011 ▸; Rath *et al.*, 2014 ▸), spectro-temporal characterization of XUV pulses (Ding *et al.*, 2021 ▸) and stimulated X-ray emission (Kroll *et al.*, 2020 ▸) have also been reported. In recent years the technique of two-color pump/probe experiments was utilized to reveal the non-equilibrium dynamics in diamond (Inoue *et al.*, 2016 ▸) and magnetite (Pontius *et al.*, 2018 ▸). The investigation of the temporal dynamic of systems is key to the fundamental understanding of its underlying physics. Special set-ups offer the possibility to provide two XUV beams directly within the FEL with a limited temporal delay range up to 1 ps (Allaria *et al.*, 2013 ▸; Hara *et al.*, 2013 ▸; Marinelli *et al.*, 2015 ▸). A different approach is the use of a split-and-delay unit (SDU). These devices provide a large temporal delay range combined with a high temporal stability (Sorgenfrei *et al.*, 2010 ▸; Wöstmann *et al.*, 2013 ▸; Osaka *et al.*, 2016 ▸; Lu *et al.*, 2018 ▸).

Until 2020 at FLASH2 only one SDU was permanently installed, integrated within the reaction microscope endstation (REMI) at beamline FL26 which specializes in atomic, molecular and optical physics and is provided by the Max Planck Institute for Nuclear Physics (Schmid *et al.*, 2019 ▸; Meister *et al.*, 2020 ▸). It offers time-resolved experiments with a temporal delay of ±2500 fs and delivers a high total transmission of *T* > 0.7 for photon energies below *h*ν < 150 eV. Currently FLASH2 offers FEL beams with photon energies in the fundamental up to *h*ν ≃ 300 eV, and in the future up to *h*ν ≃ 540 eV as planned in the FLASH2020+ project (Rönsch-Schulenburg *et al.*, 2019 ▸). Taking the third harmonic of the radiation into account, X-ray pump/X-ray probe experiments even at a photon energy of *h*ν = 1620 eV can then be performed.

With the commissioning of the Free-Electron LASer in Hamburg 2 (FLASH2) the use of variable-gap undulators was introduced (Faatz *et al.*, 2016 ▸), enabling new experimental possibilities for users, *i.e.* the flexibility to change the photon energy of the FEL beam within a few seconds (Faatz *et al.*, 2017 ▸). Further, operating the undulators in two groups allows two almost independently choosable, distinct photon energies to be generated (Schneidmiller *et al.*, 2019 ▸). Future sub-femtosecond pulses (Beye *et al.*, 2020 ▸) are of particular importance to elucidate the electronic dynamics associated with inner-shell excitations. To fully explore the new possibilities requires an instrument which allows the aforementioned features of FLASH2 to be combined with the possibility to perform time-resolved experiments.

To enable such experiments with high flexibility, an SDU has been built and permanently implemented at FLASH2, as described in this paper. It is located in the Kai Siegbahn hall, with the nominal distance of its first mirror to the source being 61 m. It serves beamline FL24 with focused and unfocused open ports as well as beamline FL23 to perform time-resolved pump/probe experiments in the XUV and soft X-ray regions. A major feature of the presented SDU is that, in principle, it does not affect the FEL beam except in the introduced delay. It further allows the use of the SDU in combination with other instruments of the beamline, *e.g.* optical pump beam, and the users’ own analysing devices at the open port, *e.g.* spectrometers.

## 2. Concept and design of the SDU

### 2.1. Optical set-up

The SDU was designed with the demand to cover the whole envisioned spectral region of FLASH2 with a reasonable transmission. As SDUs based on amplitude splitting suffer from high absorption within the utilized materials in the XUV and soft X-ray regime (Haga *et al.*, 1998 ▸; Hilbert *et al.*, 2013 ▸), the SDU presented here relies on geometric wavefront splitting at a sharp edge of a beam splitting mirror at grazing incidence angles.

**Figure 1:**
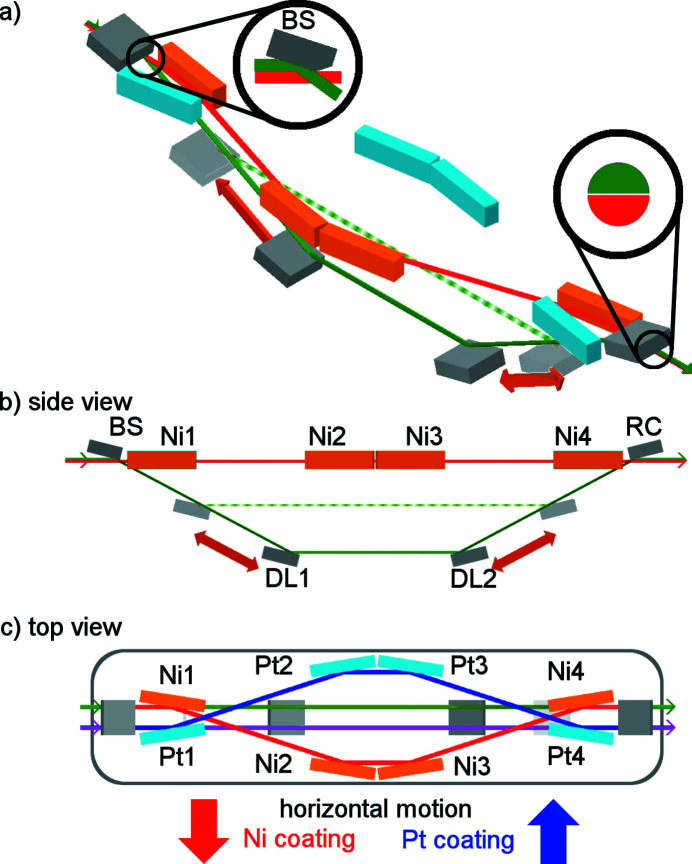
Schematic drawing of the SDU showing FEL beams utilizing the Ni-coated mirrors (orange). The initially top section of the beam (green) is reflected by the beam splitter mirror (BS) downwards into the variable delay path (gray mirrors). The mirrors DL1 and DL2 can be moved along the beam path as indicated by the orange arrows and induce a temporal delay into this beam. The initially lower section of the FEL beam (red) passes the BS unreflected (see top inset) and propagates in the fixed delay path (Ni1–Ni4, orange mirrors). Both beams are recombined at the recombining mirror (RC, see lower inset). A small gap between both beam parts remains after leaving the SDU. The optical bench [gray frame, in (*c*)] can be moved horizontally to change the positioning from the Ni- to the Pt-coated mirrors Pt1–Pt4 (light blue). In the variable delay path the Ni and Pt coatings are evaporated side-by-side on the mirrors BS, DL1, DL2 and RC.

The conceptual design is shown in Fig. 1 ▸. The first mirror (BS), as the beam splitter, separates the top part of the beam (green) from the lower part (red) by reflecting it downwards [see upper-left inset in Fig. 1 ▸(*a*)]. The lower part of the FEL beam propagates unaffected below BS into the fixed beam path. It is reflected by the mirrors Ni1–Ni4 at a grazing incidence angle of ϑ_f_ = 1.3° in the horizontal plane, as shown in Fig. 1 ▸. The mirrors are arranged in a symmetrical order, thus after passing the SDU the beam leaves the SDU collinear to its original direction. The beam is directed onto the experiment passing close to the cutting edge of the recombining mirror (RC). The nominal distance between Ni1 and Ni2 (and between Ni3 and Ni4) is *l*_f_ = 2075 mm. The nominal distance between Ni2 and Ni3 is 554 mm.

The downward reflected beam is directed into the variable delay path which has been designed at a grazing incidence angle of ϑ_v_ = 1.8°. As shown in Fig. 1 ▸(*b*), this beam is reflected at the mirrors DL1, DL2 and RC. As indicated by the orange arrows, the positions of DL1 and DL2 can be adjusted to set the distance between BS/DL1 and DL2/RC in the range *l*_v_ = 700–2450 mm. The beam is reflected on the lower edge of RC toward the experiment. RC acts as the counterpart of BS recombining both beams except for a narrow gap between them – see right-hand inset in Fig. 1 ▸(*a*). As the beam is split horizontally, both partial beams will be separated vertically in the far field. This enables the SDU to be combined with other techniques, *e.g.* spectrometry, analyzing both beams simultaneously after the interaction. On a spectrometer detector the beams are separated in the vertical plane while the respective signals can be resolved in the horizontal plane. The SDU is moved into the beam for operation. Via the vertical positioning of the optical bench the splitting ratio of both partial beams is set.

The distance between the cutting edge of BS and the cutting edge of RC amounts to *d*_total_ = 5400 mm. This long distance is a consequence of the requested delay range of up to +18 ps for a grazing angle of ϑ_v_ = 1.8°. As the mirrors DL1 and DL2 can be moved along the beam path, a change of the distance *l*_v_ leads to a change of the optical path length in the variable beam path. The difference in time delay between both beams Δ*t* can be calculated as

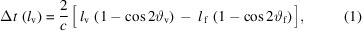

where *c* is the speed of light, *l*_v_ the distance between BS and DL1 at a glancing angle of ϑ_v_, and *l*_f_ the distance between Ni1 and Ni2 at a glancing angle ϑ_f_. With the aforementioned values a nominal absolute time delay of Δ*t*_v_ = 9.22–32.25 ps in the variable delay path and Δ*t*_f_ = 14.25 ps in the fixed delay path with respect to the direct beam is added. This results in a delay range of −5 ps < Δ*t* < +18 ps for the present SDU. For Δ*t* < 0 ps, the ray from the variable beam path hits the experiment first, pumping the sample under investigation, and accordingly the ray from the fixed beam path acts as the probe. For Δ*t* > 0 ps the beam from the fixed beam path is used to pump the sample and the beam from the variable beam path probes the sample under investigation.

For an increase of the total transmission at high photon energies (*h*ν > 800 eV) the beam paths can be switched to Pt-coated mirrors by moving the whole optical bench with all optical components horizontally and under vacuum – see Fig. 1 ▸(*c*). In this set-up the mirrors Pt1–Pt4 (light blue) form the fixed delay path (blue), again under ϑ_f_ = 1.3° glancing angle. For the variable delay paths the Ni and Pt coatings are evaporated side by side on the same set of mirrors (BS, DL1, DL2, RC). Therefore, the variable delay path with Pt-coated section of the delay mirrors (magenta) lies parallel to the delay path with the Ni-coated section (green). This results in a total of 12 mirrors providing four beam paths with four reflections each.

**Figure 2:**
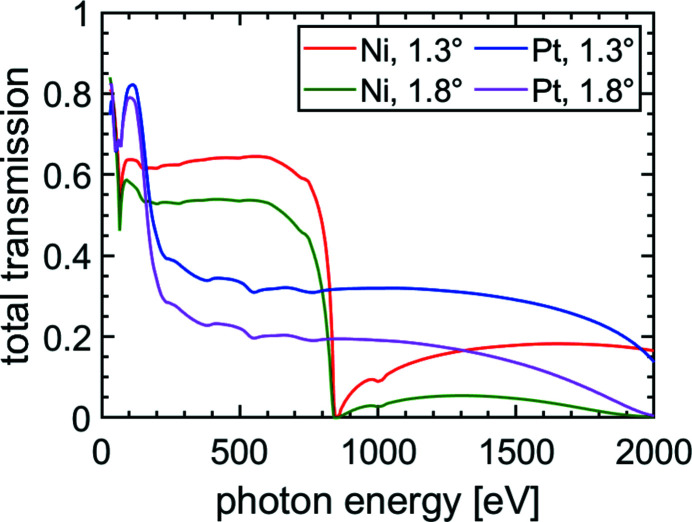
Calculated total transmission of the mirror coatings used in the different beam paths based on CXRO (Henke *et al.*, 1993 ▸), assuming a FEL beam *p*-polarized in the horizontal plane. The grazing incidence angle ϑ_v_ = 1.8° is associated with the variable beam path, and a grazing incidence angle of ϑ_f_ = 1.3° is applied in the fixed beam path. Note that only the same type of mirror coating [Ni (red and green) or Pt (blue and magenta)] can be chosen simultaneously.

Fig. 2 ▸ shows the calculated transmissions in the individual beam paths based on CXRO (Henke *et al.*, 1993 ▸) for a horizontal linear polarization of the FEL beam. The variable beam path with Ni coatings shows a total transmission of *T*_Ni,v_ > 0.50 up to photon energies of *h*ν = 650 eV. This covers the future maximum photon energy of *h*ν = 540 eV reachable by FLASH2 in the fundamental (Rönsch-Schulenburg *et al.*, 2019 ▸). Due to its shallower grazing incidence angle of ϑ_f_ = 1.3°, the total transmission of the fixed beam path of *T*_Ni,f_ > 0.60 is noticeably higher in this spectral region. Furthermore, the total transmission up to *h*ν = 800 eV for the respective beam paths amounts still to *T*_Ni,f_ > 0.45 and *T*_Ni,v_ > 0.30. Approaching the Ni *L*-edge at *h*ν = 852.7 eV, the total transmission decreases noticeably. To complement the performance of the SDU at high photon energies, a second Pt-coated beam path is implemented. The Pt-coated mirrors cover the energy region 800 eV < *h*ν < 1800 eV, providing a transmission of *T*_Pt,v_ > 0.13 and *T*_Pt,f_ > 0.29 for photon energies of *h*ν < 1500 eV. For higher photon energies, at least *T*_Pt,v_ > 0.06 in the variable beam path is achieved which is sufficient to provide a probe beam in most cases. The total transmission in the fixed delay path is *T*_Pt,f_ = 0.23 at *h*ν = 1800 eV. Given this fact, even odd harmonics of the fundamental FEL beam can be transmitted with reasonable intensity in soft X-ray pump/soft X-ray probe experiments from the XUV beyond the soft X-ray region. Additionally, the Pt-coated mirrors offer a second possibility for an increased reflectivity for photon energies below *h*ν ≃ 150 eV. Experimentally the total transmission of the Ni coating was measured to *T*_v_ = 0.47 and *T*_f_ = 0.54 at *h*ν = 124 eV. This somewhat lower transmission than calculated originates from losses at the beam splitter edge, *e.g.* optical refraction, which has not been taken into account in the calculations. As the presented total transmission of each beam path covers a broad spectral range, the scope of application of the SDU is not restricted to pump/probe experiments with only one photon energy. With a two-color operation of the FEL (Schneidmiller *et al.*, 2019 ▸) and proper selection of thin filters in the respective beam paths, two-color pump/probe experiments are possible where the optical pump and optical probe do not share the same photon energy.

**Figure 3:**
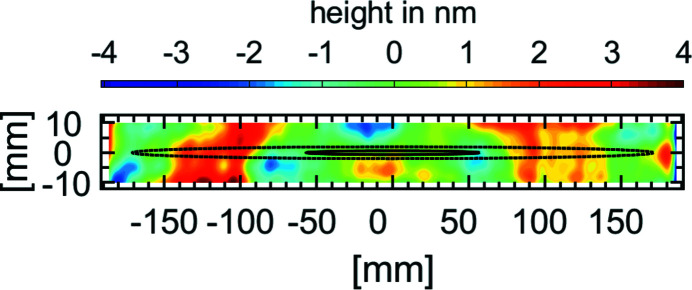
Surface profile of the Ni1 mirror. Characterized is the clear aperture of 370 mm × 20 mm. The typical profile results in a concave radius of above 500 km and a slope error of 0.032 arcsec (r.m.s.) tangential and 0.038 arcsec (r.m.s.) sagittal. The 2σ and 6σ footprints of a FEL beam at 150 eV are drawn for reference according to Kuhlmann & Plönjes (2013 ▸).

The mirror sizes were designed for the estimated divergences presented in 2013 (Kuhlmann & Plönjes, 2013 ▸). Based on this paper, for a photon energy of *h*ν = 150 eV the calculated spot size for a Gaussian profile at the entrance of the SDU amounts to Ξ = 8 mm (6σ) (3 mm FWHM). Given the geometrical design, the effective aperture of the SDU is limited by the size of the mirrors which are given in Table 1 ▸. The dimensions of the Ni mirrors in the fixed delay beam path are 380 mm × 25 mm and for the Pt mirrors are 250 mm × 25 mm. In the fixed delay paths the nominal horizontal clear aperture amounts to 8.4 mm for the Ni-coated mirrors and 5.4 mm for the Pt-coated mirrors. For all mirrors of the fixed delay paths the nominal vertical clear aperture amounts to 20 mm. Notice that just the lower half of the vertical clear aperture of the mirrors is used in the fixed delay path. As indicated in Fig. 3 ▸, the 6σ footprint of a FEL beam at *h*ν ≥ 150 eV is included in the mirror surface. In the 2σ footprint the included peak-to-valley value amounts to Δ*s* < 2.5 nm at 150 eV. For lower photon energies the 6σ beam diameter surpasses the aperture of the mirrors and the beam cannot be covered by the mirrors entirely. Given the mirrors in the variable beam path with a size of 250 mm × 50 mm, a nominal vertical clear aperture of 7.5 mm follows. When cutting the beam in the middle the BS mirror includes nearly the whole top half 6σ-footprint of this beam. According to the assumed divergences this holds true down to *h*ν = 70 eV. The nominal horizontal aperture of the variable beam path is given by the width of the applied coatings of the mirror which is 30 mm for the Ni coating and 12 mm for the Pt coating, and thus entails the entire beam over the whole spectral range.

The X-ray mirrors were manufactured and characterized by Carl Zeiss SMT GmbH before application of the coatings. The reflective coatings were applied by the Fraunhofer Institute for Material and Beam Technology IWS. An exemplary height profile of the Ni1 mirror is shown in Fig. 3 ▸. For this mirror, the radius of curvature was determined by a Carl Zeiss D100 Direct measuring interferometer to over 500 km along the tangential mirror axis with a slope error below 0.032 arcsec (r.m.s.). In the sagittal plane the slope error amounts to 0.038 arcsec (r.m.s.). As can be depicted from Fig. 3 ▸, the maximal peak-to-valley value is 8.1 nm. The roughness has been measured by a DI Nanoscope D3100M to below 0.3 nm (r.m.s.) and confirms the excellent quality of the mirrors for applications with XUV and soft X-ray beams under grazing angles. The other Ni-coated mirrors show a similar profile with tangential slope errors of below 0.03 arcsec (r.m.s.). The tangential and sagittal slope of the variable beam path mirrors and the Pt-coated mirrors is below 0.02 arcsec (r.m.s.) and 0.04 arcsec (r.m.s.), respectively. The minimum radius of these mirrors is over 300 km and the maximal peak-to-valley value is below 7.1 nm. The beam splitting edge of the mirrors BS and RC were cut at 10 mm from the end of the respective mirror, reducing the clear aperture of these mirrors.

### 2.2. Technical realization

**Figure 4:**
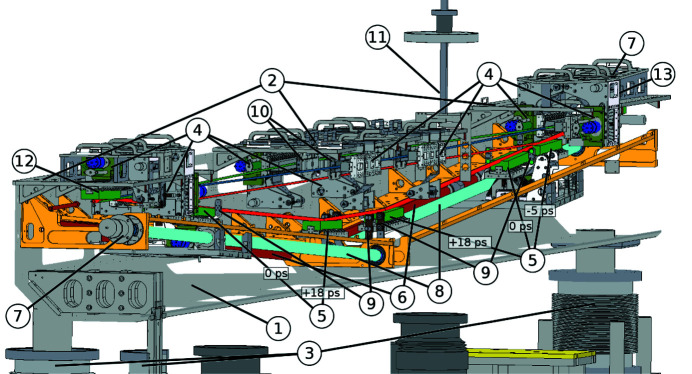
Mechanical structure of the SDU. (1) Optical bench, (2) base metal plates, (3) moving base with vacuum bellows, (4) mirror mountings, (5) DL1 and DL2 at −5 ps, 0 ps and +18 ps positions, (6) guiding rails, (7) UHV-compatible stepper motors of the delay stage, (8) ball screw drives, (9) two beam interferometer, (10) relative pulse intensity measurement device, (11) retractable blocking stages with filters, (12) beam splitter BS, (13) recombination mirror RC.

A technical drawing of the SDU is shown in Fig. 4 ▸. All parts are made, as far as feasible, out of stainless steel to avoid strain within the SDU which may be caused by temperature driven expansions. The optical bench (1) consists of a single sheet of stainless steel with a thickness of 4 mm. The upper part of the optical bench is connected via metal plates (2), increasing the stiffness and serving as platforms for the mirror holders of BS, RC and for the fixed beam path. The mechanical contact of the optical bench has been decoupled from the vacuum chamber in order to avoid any bending of the optical bench when the vacuum chamber is evacuated. Bellows (3) allow the optical bench to be moved vertically and horizontally as a whole. The optical bench rests on top of two granite blocks which are decoupled from vibrations of the ground floor by vibration isolating damping elements. There is no additional treatment of anti-vibrational mechanisms of the floor of the beamline and the SDU. An improved vibrational isolation can be achieved in future by the insertion of active damping elements.

The mirrors are attached on mirror holders (4) which allow an adjustment of the pitch and roll angles with high accuracy (Noll, 2003 ▸; Wöstmann *et al.*, 2013 ▸). A gimbal mount is realized by braces with flexible joints which face towards the center of the mirrors’ surface. Hence, all three pivot points are located within the mirror surface with a low parasitic movement of the mirror. Every mirror holder is equipped with two UHV-compatible stepper motors with gear units which allow an *in situ* movement of the pitch and roll axes with respect to the beam. 200 motor steps correspond to one revolution of the motor, which in turn deflects the pitch angle by 25 µrad and results in a lateral movement of 625 µm of the unfocused beam at the sample position at a distance of 25 m. The two last mirrors of every beam path (Ni4, Pt4 and RC) are dedicated to adjusting the position and overlap of the partial beams at the experiment. The mirrors DL1, DL2, RC, Ni4 and Pt4 are equipped with piezoelectric actuators. This allows the pitch angle to be set at a nominal accuracy of δϑ = 38 nrad in the range Δϑ = 1250 µrad. The yaw axis around the surface normal is of minor importance and was therefore aligned manually before installation of the SDU via fine-thread screws. All movable parts are equipped with limit switches to prevent accidental damage to any parts.

The variable mirrors DL1 and DL2 [see (5) in Fig. 4 ▸] are both placed on sleds which move on guiding rails (6) along the variable beam path. The guiding rails are connected to the optical bench via corner profiles. Every sled is driven individually, but identically, by a stepper motor (7) via a ball screw drive (8). The positions of the variable mirrors are measured by optical encoders with a nominal division of δ*l*_v,Enc_ = 5 µm of the encoder marker, corresponding to a temporal resolution of δ*t* = 66 as. While moving, the pitch angle of the mirrors is measured via an optical interferometer (9; Attocube, IDS3010), and is automatically corrected in a closed feedback loop via the piezoelectric actuators.

**Figure 5:**
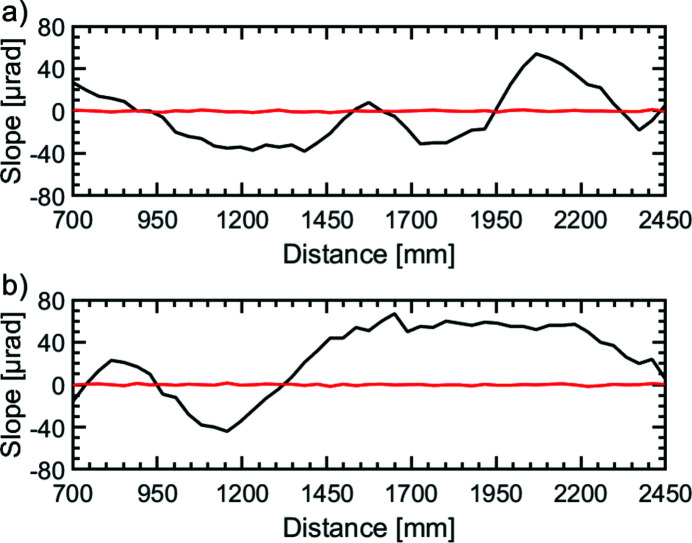
Slope deviation of the mirrors (*a*) DL1 and (*b*) DL2 over the delay range as measured by the interferometric system. The peak-to-valley value of the slope deviation amounts to Δϑ = 80 µrad for DL1 and Δϑ = 165 µrad for DL2 over the whole delay path. The angular deviation of the beam is compensated by the closed feedback interferometer system acting on the mirrors, yielding a correction precision of δϑ = 0.66 µrad (r.m.s.) (red line, see text).

As both delay mirrors are moved independently on guiding rails over a distance of Δ*l*_v_ = 1.75 m, mechanical inaccuracies are inevitable. These arise from reproducible errors from not perfectly linear guiding rails as well as non-reproducible errors like mechanical inaccuracies of the utilized bearings. A laser was applied parallel to the delay stage at the height of the central mirror surface of the respective delay mirror. The mirror was replaced by a CCD camera (Basler, acA645-gm100) with a pixel size of 9.9 µm which measured the height deviation of the sled when moving along the delay stage. As these measurements were performed in a cleanroom tent under ambient flow conditions the fluctuations were noticeable. The averaged height deviation does not exceed Δ*h* = 8 µm and Δ*h* = 25 µm for the delay stages of DL1 and DL2, respectively. As the angular deviation has a greater impact on the stability of the system, the angular deviation has been measured over the delay range by the optical interferometer. The results are shown in Fig. 5 ▸. The peak-to-valley value for DL1 shows a maximum deviation of Δϑ = 92 µrad and for DL2 of Δϑ = 111 µrad. Since both maximum deviations are within the provided correction range of the closed-feedback interferometric system of Δϑ = 1250 µrad, those errors are corrected automatically with a precision of δϑ = 0.66 µrad (r.m.s.) (red lines in Fig. 5 ▸) by the piezoelectric actuators.

As can be seen in equation (1), the accuracy of the delay setting directly depends on the precise positioning of the sleds. Therefore, the reproducibility of the motorized delay stage was verified by the use of the optical encoder and the interferometer. The motorized delay stage was moved for a certain amount of motor steps and the position of the sled was simultaneously and independently measured by the optical encoder and the optical interferometer. For 20000 motor steps, corresponding to a distance of Δ*l*_M_ = 5 mm or a nominal delay of Δ*t* = 66 fs, the optical encoder measured a distance of Δ*l*_E_ = 4.998 ± 0.003 mm while the interferometer measured a distance of Δ*l*_I_ = 5.000 ± 0.001 mm. For a movement of 200000 motor steps, corresponding to a nominal distance of Δ*l* = 50 mm or Δ*t* = 660 fs, the optical encoder measured a real distance of Δ*l*_E_ = 49.987 ± 0.003 mm while the interferometer specified Δ*l*_I_ = 49.989 ± 0.003 mm. In both measurements the mirror travel distances determined by the external devices of optical encoder and interferometer are in good agreement with each other. The relative difference between the encoder position and the interferometer position amounts to 0.04% or δ*t* = 26 ± 40 as in this example. In every single case, the differences of the optical encoder and the interferometer system did not exceed the nominal resolution of the optical encoder of δ*l*_v,Enc_ = 5 µm. Therefore, the mechanical realization ensures a temporal resolution of better than δ*t* = 66 as.

The optical bench is placed into a vacuum chamber with a total length of 6 m which is pumped by three ion-getter pumps (Agilent, Vacion Plus 500 Diode, 500 l s^−1^ each) to maintain a vibration-free vacuum at about 5.0 × 10^−9^ mbar. To reach the working pressure of the ion getter pumps, a turbomolecular pump (Pfeiffer Vacuum, HiPace700, 685 l s^−1^) is installed which is mechanically isolated from the vacuum chamber, and is turned off after pump down to avoid vibrations.

### 2.3. Beam manipulation and diagnostic

**Figure 6:**
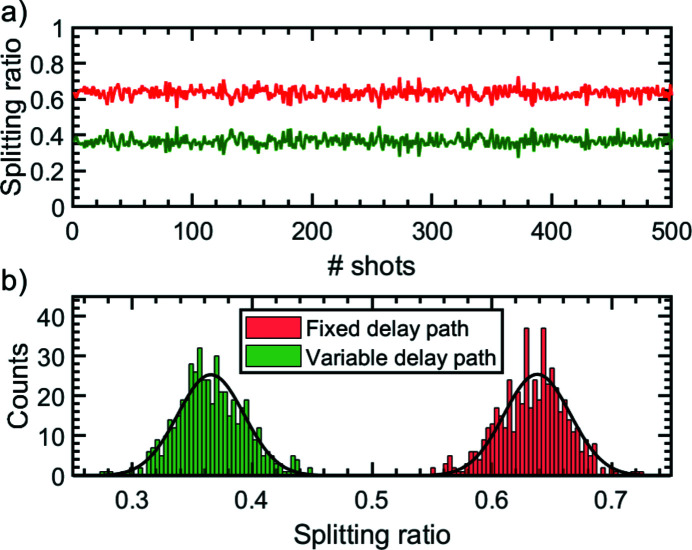
(*a*) Measured splitting ratio variations for an average splitting ratio of 63:37 over 500 shots, taken at a rate of 1 Hz. (*b*) Splitting ratio variation of both partial beams. The splitting ratio variation amounts to ±0.033 (FWHM) and 0.029 (r.m.s.), respectively.

**Figure 7:**
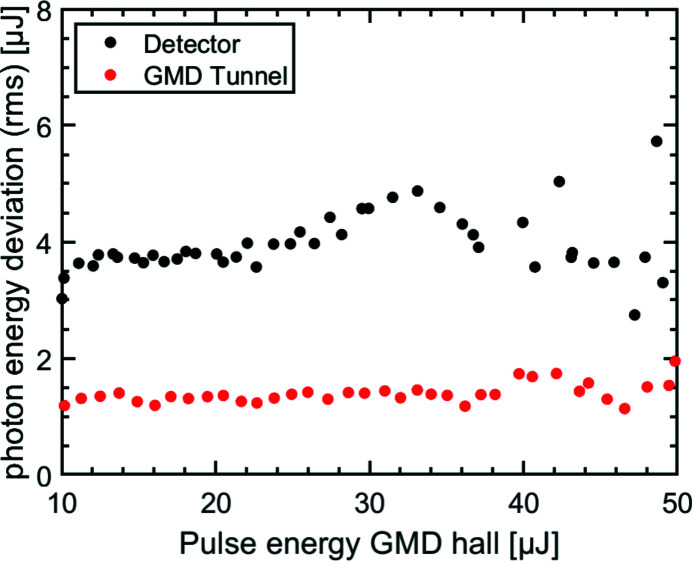
Calibration measurement of the relative pulse intensity measurement device (PATIM detector) at a wavelength of λ = 8.5 nm on a shot-to-shot basis with respect to the GMD in the hall. Over the considered pulse energy range, the absolute energy deviation is δ*E*_pulse_ ≃ 4 µJ (r.m.s.). The pulse energy measured by the tunnel GMD is simultaneously obtained and given as a reference with δ*E*_pulse_ < 2 µJ.

The intensity ratio of the two partial beams can be chosen according to the needs of the experiment by moving the optical bench in the vertical direction. In Fig. 6 ▸(*a*) for a nominal splitting ratio of 63:37 the fluctuations of this ratio are shown on a shot-to-shot basis for 500 shots taken at 1 Hz. Fig. 6 ▸(*b*) shows a histogram of these fluctuations. In this particular case the splitting ratio is varying by 0.029 (r.m.s.) and ±0.033 (FWHM), respectively. These fluctuations arise in particular from the pointing of the FEL beam leading to a varying splitting of the footprint at the beam splitting edge. *In situ* single-shot intensity measurements are important for pump/probe experiments. To enable this, the mirrors Ni2 and Pt2 are equipped with microchannel plate detectors which measure the emitted photoelectrons of the mirror surface with intra-bunch single-pulse resolution (so-called PATIM) – see Fig. 4 ▸ (10). With the intensity data provided by both gas monitor detectors (GMDs) in the hall and in the tunnel (Tiedtke *et al.*, 2008 ▸), this allows the proportion of the beam intensity passing the fixed delay beam path to be calculated. In Fig. 7 ▸ a calibration measurement taken at a wavelength of λ = 8.5 nm is depicted. This shows the pulse energy deviation of the signal with respect to the GMD located in the hall. Over the scanned pulse energy range up to *E*_pulse_ = 50 µJ an accuracy of about δ*E*_pulse_ ≃ 4 µJ (r.m.s.) with respect to the GMD hall is achieved. As reference, the pulse energy deviations of the GMD detector in the tunnel are shown in red, which show a pulse energy deviation of <2 µJ (r.m.s.).

Within every beam path, a retractable blocking stage – see Fig. 4 ▸ (11) – is available which allows the individual beam path to be blocked to check beam positions separately. The blocking stages can be equipped with filter windows in order to separate distinct photon energies of the FEL, *e.g.* the fundamental from a harmonic. With this option, the SDU enables time-resolved two-color pump/probe experiments. Furthermore, the filter stage of the delay path provides a Ce:YAG screen for beam position monitoring. Another imaging stage is located right behind the SDU in a distinct vacuum chamber. With this imaging stage the recombined partial beams can be imaged via a Ce:YAG screen. Additionally, this monitoring chamber provides another stage with interchangeable filters.

## 3. Performance

### 3.1. Basic alignment

**Figure 8:**
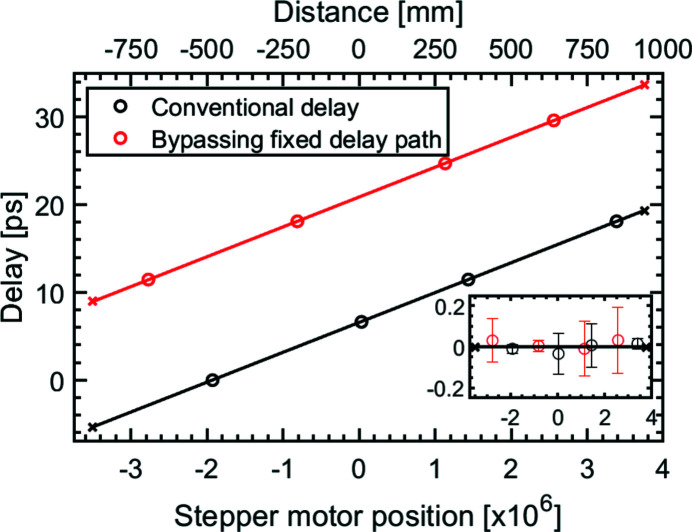
Linearity of the delay stage as measured by a laser with reduced coherence time. The black circles refer to an interference of the two beams in the fixed delay path and variable delay path. The red circles refer to the measured interferences between the beam of the variable delay path and a beam passing directly through the SDU. The additional delay obtained by bypassing the fixed delay path amounts to 14.3 ps. The covered delay range of the SDU spans between the crossed markers, allowing delays between −5 ps and 18 ps with the conventional set-up and up to 32 ps when bypassing the fixed delay path. The fit results in a slope of (2.95 ± 0.02) × 10^5^ steps of the stepper motor for 1 ps. In the inset the residuals of the slopes are shown, which amounts to 6 fs. The error is given by the coherence length of the individual coherence signals.

A prealignment was carried out with an optical laser at λ = 660 nm with a reduced coherence length of the order of 300 µm (Schäfter + Kirchhoff, 51nano-FCM) to show the linearity of the delay stage. A special feature of this optical laser is that it exhibits additional coherence signals at temporal intervals of Δ*t* = 6.6 ps, 11.5 ps, 18.1 ps, 24.7 ps and 29.6 ps. The SDU was inserted partially into the optical beam leaving one part unaffected. In this case, two coherence measurements were examined simultaneously, see Fig. 8 ▸: one interference measurement of the partial beams of the fixed delay path and the variable beam path (black circles), and one interference measurement of the direct beam and the variable delayed beam (red circles). The known temporal distances of the coherence signals are assigned to the lateral positions of the delay stage leading to a linearity calibration of the delay stage. The resulting slope gives a ratio of 1 ps per (2.95 ± 0.02) × 10^5^ stepper motor steps. This corresponds to a reflection angle of ϑ = 1.83° which is in the design range of the reflection angle of ϑ_v_ = 1.8°. Given the mechanical constraints, a delay range of −5 < Δ*t* < +18 ps is achieved. According to the slope the difference between the fixed delay path and the direct beam amounts to 14.3 ps. Bypassing the fixed delay path gives the opportunity to extend the achievable delay range up to 33 ps.

### 3.2. Temporal stability

As the temporal jitter is the temporal deviation of the given delay between both beams, interference fringes offer an opportunity to evaluate this characteristic of the SDU. A change of time delay between both beams results in a movement of the observed interference fringes. Considering only one local point of the whole interference pattern this movement corresponds to a change of intensity *I*_Exp_ at this local point. By evaluating the intensity at this point at a nominally fixed delay position the actual time delay between both partial beams can be obtained. With the minimum and maximum intensity of the interference fringes (*I*_max_, *I*_min_) the time delay δ*t* can be calculated by

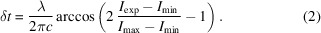

**Figure 9:**
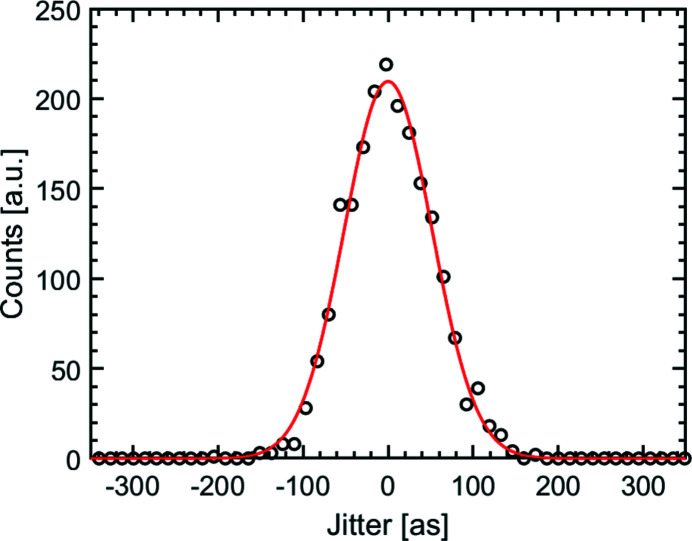
Jitter histogram of the SDU derived from interference measurements with visible light at 633 nm. A Gaussian profile with a width *t*_j_ = 121 ± 2 as (FWHM) has been fitted to the data. 2000 data points were taken with a sample period of 80 Hz, *i.e.* over a measurement time of 25 s.

**Figure 10:**
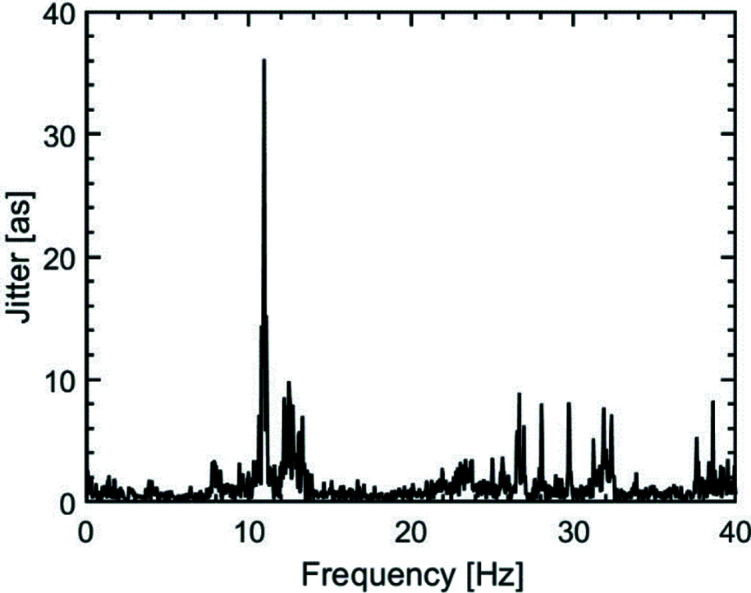
Fast Fourier transform of the interference measurements with visible light. The SDU with dampers shows a prominent resonance at 11 Hz arising from the incoupling of ground vibrations from the hall.

It shall be emphasized here that, due to the ambiguity of the arccos function, the time delay can only be examined unambiguously for values of *I*_min_ < *I*_Exp_ < *I*_max_. Therefore, it is reasonable to measure between two interference fringe maxima and to use a laser with a long wavelength, because that enlarges the measurable delay jitter. An optical laser with a wavelength of λ = 633 nm was used to create suitable interference patterns. The interference pattern was measured with a camera (Basler, acA720-290gm) at a distance of *L* = 24 m from the RC. As the detector is placed perpendicular to the main propagation direction of the beams, the impact of small vibrational movements of the camera on the measured interference fringes is negligible. The data were taken at a rate of 80 Hz. The temporal jitter of the SDU is shown in Fig. 9 ▸. The resulting time delays of the data are binned in count rates by intervals of 10 as and shown as a histogram. A Gaussian profile is fitted to the histogram, indicating a timing jitter of *t*_j_ = 121 ± 2 as (FWHM). In Fig. 10 ▸, the fast Fourier transform of the temporal jitter is shown. A main resonance of the optical bench at 11 Hz is observed, probably resulting from ground floor vibrations.

### 3.3. Pointing stability

**Figure 11:**
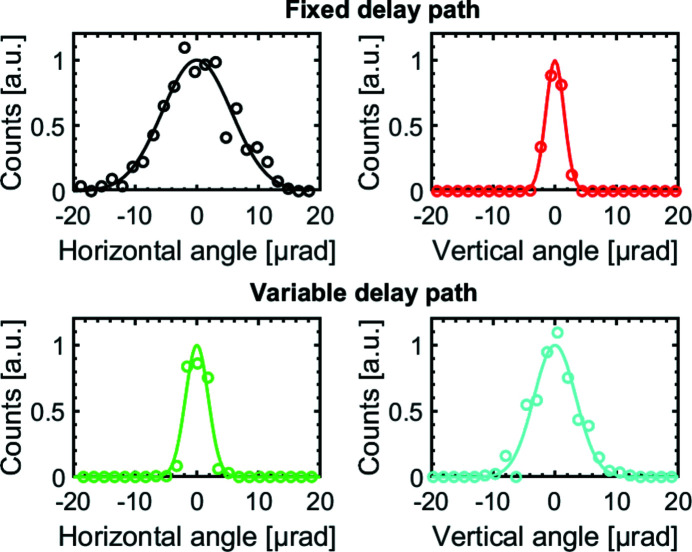
Pointing stability of both Ni-coated beam paths in the horizontal and vertical axis. In the fixed delay path a pointing stability of δ*u*_f,horizontal_ = 6.5 µrad (FWHM) and δ*u*_f,vertical_ = 2.8 µrad (FWHM) was measured. For the variable delay path it amounts to δ*u*_v,horizontal_ = 4.3 µrad (FWHM) and δ*u*_v,vertical_ = 9.7 µrad (FWHM).

To measure the pointing stability of the SDU, both partial FEL beams at λ = 10 nm were set on separate positions on an XUV-sensitive CCD camera (Princeton Instruments, PIXIS-XO:2KB). After passing the Ni-coated beam path, the spot positions were recorded on a shot-to-shot basis over 500 shots at a distance of *L* = 24 m. The beam size was reduced by inserting an aperture with a diameter of 2 mm at a distance of 13 m in front of the SDU. The resulting angular pointing stability is shown in Fig. 11 ▸. The pointing stability of the fixed beam path amounts to δ*u*_f,horizontal_ = 6.5 µrad (FWHM) in the horizontal plane and δ*u*_f,vertical_ = 2.8 µrad (FWHM) in the vertical plane. The pointing stability of the variable delay path in the horizontal plane is δ*u*_v,horizontal_ = 4.3 µrad (FWHM) while in the vertical plane it shows δ*u*_v,vertical_ = 9.7 µrad (FWHM). The difference in the pointing stability in the respective pitch and roll axis of the mirror mounts is attributed to the reversed angular orientation of the mirror mounts in these beam paths. The variable beam path is affected more by vibrations in the vertical direction introduced from the ground floor into the SDU. The footprint of an unfocused beam at the sample position is of the order of 10 mm and the displacement from pointing of the variable beam path at this position amounts to 0.2 mm (FWHM) in the vertical direction. The overlap between both partial beams of the SDU is thus maintained. The pointing of the FEL in this set-up was determined to 1.5 µrad. As a comparison, an angular pointing measurement before decoupling the SDU from external vibrations was taken. In this measurement the pointing stability is lowered to δ*û*_f,horizontal_ = 13.2 µrad (FWHM) and δ*û*_f,vertical_ = 3.5 µrad (FWHM) in the fixed beam path and is decreased slightly to δ*û*_f,vertical_ = 8.0 µrad (FWHM) in the variable delay path while no significant change was measured for δ*û*_v,horizontal_ = 4.3 µrad (FWHM).

## 4. Characterization of FEL pulses

### 4.1. Visibility measurements

When commissioning the SDU, both partial beams of the FEL operating at λ = 8 nm were overlapped directly on an XUV-sensitive CCD camera and thereby created interference fringes. At short wavelengths a small crossing angle Ω between both partial beams is necessary to resolve the arising interference patterns. Therefore, the camera was placed at a distance of *L* = 24 m from the RC mirror. The visibility was derived from these interference fringes by

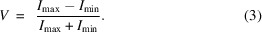

When increasing the temporal delay between both beams, the visibility is reduced due to the finite temporal coherence time of the FEL beam. From the visibility measurements the coherence time can be determined as the half width at half-maximum (HWHM) of the temporal visibility distribution of a beam. Typically, the coherence time of an FEL beam lies in the order of some femtoseconds, allowing the coherence time of the FEL pulse to be determined by means of the SDU. Likewise, a quantification of the zero-delay position with femtosecond accuracy is enabled with this set-up.

**Figure 12:**
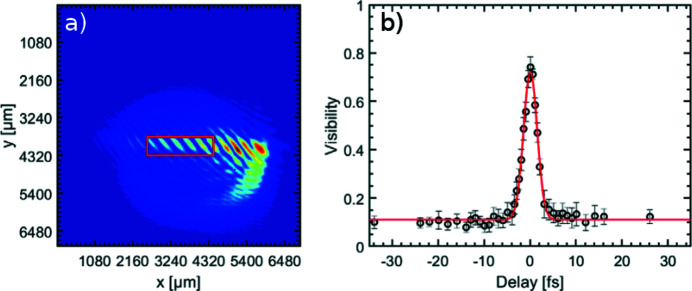
(*a*) Interference pattern taken at λ = 8 nm and Δ*t* = 0 fs. The visibility in the region marked by the red rectangle amounts to *V* = 0.8. (*b*) Temporal visibility distribution of the FEL beam at λ = 8 nm. Every point represents the average of 25 individual interference patterns taken at one delay. The resulting coherence time is τ_c_ = 1.75 ± 0.04 fs. The average visibility in the maximum was determined to *V* = 0.74 ± 0.08.

The FEL was slightly detuned on purpose leading to a significantly shorter coherence time which allows the performance of the SDU to be characterized more precisely. An exemplary interference pattern taken at λ = 8 nm and Δ*t* = 0 fs is shown in Fig. 12 ▸(*a*). Well defined interference fringes were obtained which can be distinguished from the diffraction patterns of the beam-cutting BS mirror by moving the pointing of the fixed delay beam path (bottom semicircle) slightly aside, which then leads to tilted interference fringes in the interference pattern.

From the spatial overlap *q* of both beams, the width of the interference fringes *w*, and the distance of the CCD camera to the RC mirror, the crossing angle Ω and the gap *g* between both beams at the position of the RC can be calculated. At a fringe spacing of *w* = 270 µm and an overlap of *q* = 500 µm, Ω = 30 µrad and *g* = 200 µm follow, indicating a narrow gap between both beams at the RC. For the presented shot the visibility evaluated in the area marked by the red rectangle amounts to *V* = 0.8. By changing the delay consecutively in steps of 0.5 fs and taking 25 measurements at each delay step, the delay distribution of the visibility follows, depicted in Fig. 12 ▸(*b*). A Gaussian distribution was fitted to the data yielding an average coherence time of τ_c_ = 1.75 ± 0.04 fs (HWHM). The comparatively low average visibility *V* = 0.74 ± 0.08 at Δ*t* = 0 fs is attributed to the reduced spatial coherence due to the detuning of the FEL. The obtained coherence time shows that with the SDU a temporal resolution in the sub-femtosecond timescale can be achieved as already shown by the jitter data obtained via visible light.

### 4.2. Focused beams and wavefront

**Figure 13:**
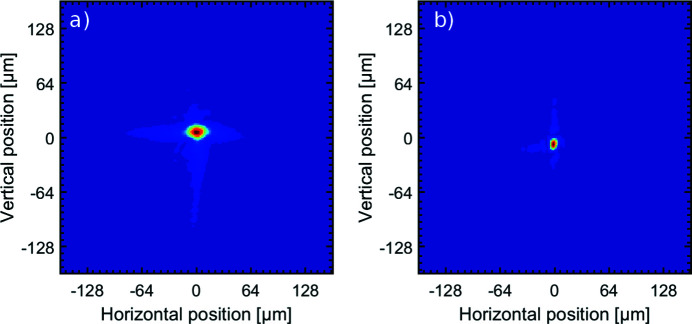
Beams in the focus position taken from backpropagation calculations with the Hartmann wavefront sensor at λ = 15 nm. The diameter of the spot size in the variable delay beam path (*a*) amounts to 19 µm (FWHM) and 14 µm (FWHM) at a focal length of 3.1 m and in the fixed delay beam path (*b*) amounts to 7 µm (FWHM) and 11 µm (FWHM) at a focal length of 3.2 m in the horizontal and vertical directions, respectively.

Beamline users typically request focused beams to perform their experiments. With the Kirkpatrick–Baez (KB) optics installed at the end of beamline FL24, both beams at λ = 15 nm were focused to a distance of 3.1 m from the KB optics. An XUV Hartmann wavefront sensor was illuminated by the diverging light behind the focus position (Keitel *et al.*, 2016 ▸). From the pattern obtained, the wavefront at the focal point was reconstructed for the beams of the fixed delay beam path and variable delay beam path individually. The result of this wavefront backpropagation calculation is shown in Fig. 13 ▸. The achieved spot size for the beam of the variable delay beam path amounts to 19 µm (FWHM) and 14 µm (FWHM) in the horizontal and vertical directions, respectively. For the fixed delay beam path the backpropagation calculation yields a spot size of 7 µm (FWHM) and 11 µm (FWHM) in the horizontal and vertical directions.

## 5. Summary

A split-and-delay unit has been built for FLASH2 in the Kai Siegbahn hall and serves beamlines FL23 and FL24. The device allows users to perform pump/probe experiments with XUV and soft X-ray beams with a temporal delay of −5 ps to +18 ps at a sub-femtosecond temporal resolution. With the possibility to choose between Ni-coated mirrors and Pt-coated mirrors, the full fundamental spectral range of FLASH2 is covered as well as harmonics up to a photon energy of *h*ν = 1800 eV. The temporal jitter between both partial beams was measured via interferences at λ = 633 nm and amounts to *t*_j_ = 121 ± 2 as (FWHM). This is close to the nominal delay resolution of δ*t* = 66 as provided by the encoder and δ*t* = 13 as provided by the nominal resolution of the stepper motors. At a splitting ratio of 63:37 a splitting ratio fluctuation of the order of 0.029 (r.m.s.) and ±0.033 (FWHM) has been measured. The angular pointing stability including the pointing error of the FEL amounts to δ*u*_f,horizontal_ = 6.5 µrad (FWHM), δ*u*_f,vertical_ = 2.8 µrad (FWHM) for the fixed delay path and δ*u*_v,horizontal_ = 4.3 µrad (FWHM), δ*u*_v,vertical_ = 9.7 µrad (FWHM) for the variable delay path. In the fixed delay paths the PATIM is installed to measure the relative intensity within the beam path on a shot-to-shot basis even for pulse trains. Additionally, with implemented filter stages a variety of two-color pump/probe experiments is possible.

The functionality of the SDU is demonstrated by visibility measurements in order to examine the coherence properties of a FEL beam with reduced temporal coherence. In this case the measured coherence time was τ_c_ = 1.75 ± 0.04 fs, revealing the outstanding opportunities of the SDU to perform pump/probe experiments in the XUV and soft X-ray region with sub-femtosecond time resolution. With the KB optics at the end of beamline FL24, the beams were focused. The beam of the variable delay path was focused to a spot size of 19 µm (FWHM) and 14 µm (FWHM) in the horizontal and vertical directions, respectively, and for the beam of the fixed delay beam path a spot size of 7 µm (FWHM) and 11 µm (FWHM), respectively, was achieved.

## Figures and Tables

**Table 1:** Nominal apertures of the mirrors of the SDU for the incoming beam The clear aperture takes into account the existence of a 5 mm not specified rim at the edges of the optical surfaces.

	BS / DL1	M_Ni_1	M_Pt_1
Mirror length	240 mm / 250 mm	380 mm	250 mm
Mirror width	50 mm	25 mm	25 mm
Grazing incidence angle	1.8°	1.3°	1.3°
Vertical clear aperture	7.5 mm / 7.8 mm	20.0 mm	20.0 mm
Horizontal clear aperture	30 mm (Ni) / 12 mm (Pt)	8.4 mm	5.4 mm
